# Silencing long non-coding RNA HOTAIR exerts anti-oncogenic effect on human acute myeloid leukemia via demethylation of HOXA5 by inhibiting Dnmt3b

**DOI:** 10.1186/s12935-019-0808-z

**Published:** 2019-04-29

**Authors:** Si-Li Wang, Yun Huang, Rui Su, Yong-Yang Yu

**Affiliations:** 1 Department of Hematology, The First Affiliated Hospital of Xiamen University, No. 55, Zhenhai Road, Xiamen, 361003 Fujian People’s Republic of China; 2 Department of Clinical Medicines, Fujian Medical University, No. 1, Xuefu North Road, Fuzhou, 350108 Fujian People’s Republic of China; 3 Department of General Surgery, The First Affiliated Hospital of Xiamen University, Xiamen, 361003 People’s Republic of China

**Keywords:** Acute myeloid leukemia, Long noncoding RNA HOTAIR, HOXA5, Dnmt3b, Promoter methylation, Proliferation, Apoptosis

## Abstract

**Background:**

As an aggressive hematological malignancy, acute myeloid leukemia (AML) remains a dismal disease with poor prognosis. Long non-coding RNAs (lncRNAs) have been widely reported to be involved in tumorigenesis of AML. Here, we define an important role of lncRNA HOTAIR in AML in relation to HOXA5 methylation.

**Methods:**

Firstly, the expression of HOTAIR was examined in AML samples and cells collected. Next, gain- or loss-of function experiments were conducted in AML cells to explore the effect of HOTAIR on AML. Then, relationship among HOXA5 promoter methylation, HOTAIR and Dnmt3b was measured. Expression of HOXA5 and cell proliferation/apoptosis-related genes was also detected. A last, in vivo assay was performed to assess the tumor formation in nude mice in order to explore the roles of HOTAIR and HOXA5 in cell apoptosis and proliferation.

**Results:**

LncRNA HOTAIR was found to be upregulated in AML cells and tissues. With silencing of HOTAIR and overexpression of HOXA5, AML cell proliferation was decreased while the apoptosis was induced. Furthermore, HOTAIR was observed to recruit Dnmt3b and to increase HOXA5 promoter methylation. Moreover, silencing HOTAIR and upregulating HOXA5 were found to induce apoptosis and reduce proliferation of AML cells in vivo.

**Conclusion:**

Our findings highlight the anti-tumor ability of HOTAIR silencing in AML, suggesting that silencing HOTAIR was able to inhibit AML progression through HOXA5 promoter demethylation by decreasing Dnmt3b.

**Electronic supplementary material:**

The online version of this article (10.1186/s12935-019-0808-z) contains supplementary material, which is available to authorized users.

## Background

As the most common type of blood disease, acute myelogenous leukemia (AML) is highly prevalent in adults with a high relapse rate [1]. The incidence of AML is approximate 1.3 per 100,000 for people under 60 years old and 12.2 per 100,000 for people over 65 years old [2]. AML is characterized by a decrease in the proliferation of functional blood cells and excessive proliferation and accumulation of immature leukemic blasts [3]. AML has been reported to be caused by diverse factors, such as cytogenetic risk, age, white blood cell count, and fetal liver tyrosine kinase 3 internal tandem duplication (FLT3 ITD) [4, 5]. In spite of the improvement achieved in controlling AML, AML still leads to low overall survival rates with poor prognosis [6]. Therefore, it is in urgent need to find out more effective biomarkers for AML to strengthen the diagnosis and prognosis of AML.

It has been widely revealed that long non-coding RNAs (lncRNAs), a family of non-protein-coding transcripts with the length of over 200 nt act pivotal functions in blood diseases [7]. Several lncRNAs have been demonstrated to function in AML, including CCDC26, KCNQ1 overlapping transcript 1 (KCNQ1OT1), and homeobox antisense intergenic RNA myeloid 1 (HOTAIRM1) [8–10]. More specifically, homeobox antisense intergenic RNA (HOTAIR) serves as an underlying predictor for the relapse diagnosis and poor prognosis in patients with AML [11]. Furthermore, the highly expressed HOTAIR was suggested to be associated with the progression of AML via the regulation of cell proliferation and apoptosis [12]. Furthermore, Up-regulation of HOTAIR and methylation of HOXA5 were found during the development of breast cancer [13] and a previous study indicated that HOTAIR and homeobox A5 (HOXA5) worked together and were closely correlated to growth and metastasis of non-small cell lung cancer [14]. HOXA5, a member belonging to the HOX gene family, could modulate the differentiation of epithelial and hematopoietic cells, and HOXA5 methylation was linked to disease aggressiveness [15]. Silencing HOXA5 has been reported to reduce cell proliferation in AML patients [16]. Dnmt3b was reported to encode a DNA methyltransferase, which is involved in aberrant epigenetic changes that lead to leukemia [17]. Elevated transcript levels of DNA methyltransferase 3b (Dnmt3b) and upregulated promoter methylation of HOXA5 were found to be associated with the developmental processes of estrogen receptor negative breast tumors [18]. The present study aims to dig out the specific mechanism by which lncRNA HOTAIR and HOXA5 methylation influences the proliferation and apoptosis of AML cells.

## Materials and methods

### Ethics statement

The present study was conducted in strict accordance to the protocols approved by the Institutional Review Board of The First Affiliated Hospital of Xiamen University. Written informed consent documentations were signed by all participating patients. All animal experiments were performed in full compliance with the national principles for animal usage in research with the approval of the Animal Care and Use Committee. All animal experiments in this study were in conformity to the guidelines of management and use of local laboratory animals and approved by the Guide for the Care and Use of Laboratory Animal issued by the National Institutes of Health.

### Study subjects

A total of 90 patients diagnosed with AML in The First Affiliated Hospital of Xiamen University from May 2016 to September 2017 were enrolled in this study, including 47 males and 43 females aged from 22 to 65 years old with the median age of 45 years old. All patients met the World Health Organization (WHO) diagnostic criteria and were diagnosed by bone marrow cell morphology test including Wright-Giemsa staining, cytochemical staining [peroxidase (POX), alpha-naphthyl-acetate-esterase (ANAE), specific esterase (CE), and periodic acid-Schiff (PAS)] and immunophenotype test (flow cytometry). Patients with combined severe infection, other solid tumors or immune system diseases were excluded. Meanwhile, 30 normal human bone marrow samples were selected as experimental controls. The clinical characteristics of enrolled patients are shown in Additional file 1: Table S1.

### Cell treatment

Human leukemia cell lines (HL-60, K562, THP-1 and U937) from American Type Culture Collection (Manassas, VA, USA) were cultured in RPMI-1640 medium (Gibco, Carlsbad, California, USA) conjugated with 10% fetal bovine serum (FBS), 10 ug/mL streptomycin and 100 U/mL penicillin in an incubator (Thermo Fisher Scientific Inc., Waltham, Massachusetts, USA) at 37 °C with 5% CO_2_. Hematopoietic stem cells were isolated from normal bone marrow samples, and reverse transcription quantitative polymerase chain reaction (RT-qPCR) was conducted to measure the level of HOTAIR in HL-60, K562, THP-1, U937 cells and normal bone marrow samples [19]. Cells in the logarithmic growth stage were detached by trypsin and inoculated into a 6-well plate with a density rate of 1 × 10^5^ cells/well. When the cell confluence reached about 75%, cells were cultured with the following plasmids according to the instructions of Lipofectamine 2000 (Invitrogen Inc., Car, Cal, USA): sh-negative control (NC) (5′-CUACAACAGCCACAACGUCdTdT-3′), sh-HOTAIR-1 (sense: 5′-GAACGGGAGUACAGAGAGAUU-3′; antisense: 3′-UUCUUGCCCUCAUGUCUCUCU-5′), sh-HOTAIR-2 (sense: 5′-CCACAUGAACGCCCAGAGAUU-3′; antisense: 3′-UUGGUGUACUUGCGGGUCUCU-5′), and sh-HOTAIR-3 (sense: 5′-UAACAAGACCAGAGAGCUGUU-3′; antisense: 3′-UUAUUGUUCUGGUCUCUCGAC-5′). After culturing for 48 h, the silencing efficiency of shRNA was detected by RT-qPCR.

### RT-qPCR

Total RNA was extracted using the Trizol kit (15596026, Invitrogen Inc., Car, Cal, USA). Based on the instructions of Primescript RT reagent kit (RR047A, TaKaRa, Tokyo, Japan), RNA was reversely transcribed into complementary DNA (cDNA). RT-qPCR was then carried out using the Fast SYBR Green PCR Kit (Applied Biosystems, Carlsbad, CA, USA) on the ABI PRISM 7300 RT-PCR System (Applied Biosystems, Carlsbad, CA, USA). The reaction conditions were as follows: pre-denaturation at 95 °C for 5 min, 40 cycles of denaturation at 95 °C for 30 s, annealing, and extension at 60 °C for 1 min. Each sample was set with 3 duplicated wells. Glyceraldehyde-3-phosphate dehydrogenase (GAPDH) gene served as an internal reference. The relative expression of HOTAIR and HOXA5 between the experiment group and the control group was calculated based on the 2^−∆∆Ct^ method. The primers sequences are shown in Table 1.

**Table 1:** Primer sequence for RT-qPCR

Genes	Primer sequence
HOTAIR	F: 5′-CAGTGGGGAACTCTGACTCG-3′
	R: 5′-GTGCCTGGTGCTCTCTTACC-3′
HOXA5	F: 5′-CGCCCAACCCCAGATCTA-3′
	R: 5′-GGCCGCCTATGTTGTCATG-3′
GAPDH	F: 5′-GCCAAGGTCATCCATGACAACT-3′
	R: 5′-GAGGGGCCATCCACAGTCTT-3′

*RT-qPCR* reverse transcription quantitative polymerase chain reaction, *HOTAIR* Hox transcript antisense intergenic RNA, *HOXA5* homeobox A5, *GAPDH* glyceraldehyde-3-phosphate dehydrogenase, *F* forward, *R* reverse

### Western blot analysis

After cell collection, the cells were lysed with an enhanced radioimmunoprecipitation assay (RIPA) lysis buffer (Boster Biological Technology, Co., Ltd., Wuhan, China) containing protease inhibitor. Protein concentration was measured using a bicinchoninic acid (BCA) protein assay kit (Boster Biological Technology, Co., Ltd., Wuhan, China). The protein was separated by 10% sodium dodecyl sulfate–polyacrylamide gel electrophoresis (SDS-PAGE) and transferred onto the polyvinylidene fluoride (PVDF) membranes. After being blocked with 5% bovine serum albumin (BSA) for 2 h at room temperature, the membranes were incubated with the addition of diluted primary rabbit antibodies (Abcam Inc., Cambridge, MA, USA) against HOXA5 (ab82645, 1:500), Bcl-2 Associated X (Bax, ab32503, 1:1000), Bcl-2 (ab32124, 1:500), MCP-1 (ab9669, 1:500), cleaved-caspase3 (ab49822, 1:500), p27 (ab32034, 1:5000), and cyclin G (ab170389, 1:100) at 4 °C overnight. After being washed three times with phosphate buffered saline-Tween 20 (PBST), the membranes were incubated after the addition of horseradish peroxidase (HRP)-labeled goat anti-rabbit secondary antibody (ab205719; 1:2000, Abcam Inc., Cambridge, MA, USA) at room temperature for 1 h. Then the membranes were washed three times with PBST and detected using the enhanced chemiluminescence (ECL, EMD Millipore Corporation, Billerica, MA, USA). Gray-value quantification of bands in western blot images was performed using Image J analysis software, and GAPDH was taken as an internal reference. The experiment was repeated three times.

### Fluorescence in situ hybridization (FISH)

The location of HOTAIR in AML cells was detected by FISH according to the instructions of RiboTM lncRNA FISH Probe Mix (Red) (Guangzhou RiboBio Co., Ltd., Guangzhou, China). AML cells were cultured in 6-well plates, which were coated with coverslips for 1 d until the cell confluence reached about 80%. After that, cells were washed with phosphate-buffered saline (PBS), fixed with 1 mL of 4% paraformaldehyde at room temperature. After being treated with 2 μg/mL protease K, glycine and acetylation reagents, cells were incubated with 250 μL of pre-hybridization solution at 42 °C for 1 h. After the pre-hybridization solution was aspirated, cells were added with 250 μL of hybridization solution containing 300 ng/mL HOTAIR probe and then hybridized overnight at 42 °C. After washing with PBST three times, the cells were stained with 4′,6-diamidino-2-phenylindole (DAPI) (1:800) diluted with PBST for 5 min, rinsed with PBST three times (3 min each time) and sealed with anti-fluorescent quencher. Five different fields were selected and photographed under a fluorescence microscope (Olympus Co., Ltd., Tokyo, Japan). Each experiment was repeated three times.

### Methylation specific PCR (MS-PCR)

Based on the DNA Methylation-Gold™ Kit (D5005, Zymo Research, Irvine, CA, USA), the methylation level of the HOXA5 promoter region was measured. The primer sequences for methylation reaction were HOXA5-MD (5′-TTTAGCGGTGGCGTTCG-3′) and HOXA5-MR (5′-ATACGACTTCGAATCACGTA-3′), and the primer sequences for the un-methylation reaction were HOXA5-UD (5′-TTGGTGAAGTTGGGTG-3′) and HOXA5-UR (5′-AATACAACTTCAAATCACATAC-3′). The purified DNA was added into cytosine to thymine (CT) conversion reagent for denaturation and bisulfite conversion. Then the desulfurization and purification were conducted using a reaction column, and the purified DNA could be used for subsequent PCR reaction. The PCR reaction conditions were as follows: pre-denaturation at 95 °C for 10 min, and 35 cycles of denaturation at 95 °C for 45 s, methylation at 56 °C for 45 s, non-methylation at 45 °C for 45 s, extension at 72 °C for 45 s, and a final elongation at 72 °C for 10 min. The reaction products subsequently underwent agarose gel electrophoresis, which were then analyzed by imaging analysis. Each experiment was repeated three times.

### Dual luciferase reporter assay

HOXA5 promoter region was detected by dual luciferase reporter assay. Cells were inoculated into the 24-well plates and cultured with plasmids using Lipofectamine 2000 when cells confluence reached 60–80%. The cells were collected after 24–48 h, rinsed with PBS three times and lysed with 75 μL lysate at room temperature for 15–20 min, shaken every several min so that the cells could be completely covered with lysate. After collection of the cell lysate, luciferase activities were immediately detected based on the instructions of Dual luciferase assay kit with a luminometer (Monolight 2010; Analytical Luminescence Laboratory, San Diego, CA, USA). During the experiment, the thymidine kinase promoter-renilla luciferase reporter plasmid (pRL-TK) was used as the internal reference, the reaction system of firefly luciferase reaction system was LAR II, and the renilla luciferase reaction system was Stop&Glo Reagent. The fluorometer was preheated, and the parameters were set. Then the determination started after each delay of 2 s, with the determination time set as 10 s. After adding with 100 μL LAR II, the fluorescent tube was added with 20 μL of cell lysate. After mixing for 2–3 times with a pipette tip, the fluorometer was placed into the fluorescent tube with reading started. Firefly luciferase reading was recorded and repeated once. A total of 100 μL of Stop&Glo Reagent was added into the same tube, and mixed. Then the fluorometer was placed into the fluorescent tube to record, and this step was repeated once. Each experiment was repeated three times.

### RNA immunoprecipitation (RIP)

The binding of HOTAIR to Dnmt3b protein was detected using a RIP kit (Merck Millipore, Billerica, MA, USA). After being washed with pre-cooled and lysed with an equal volume of RIPA lysate (P0013B, Beyotime Institute of Biotechnology, Shanghai, China) in an ice bath for 5 min, the cells were centrifuged at 14,000 rpm for 10 min at 4 °C. Part of the cell extract was taken as input, and remaining cell extract was incubated with antibody for co-precipitation. A total of 50 μL magnetic beads for each co-precipitation reaction system were washed and re-suspended with 100 μL RIP Wash Buffer, and then 5 μg of following antibody was added: rabbit anti Dmnt3b (1:100, ab2851, Abcam Inc., Cambridge, MA, UK) or rabbit anti-human immunoglobulin G (IgG) (1:100, ab109489, Abcam Inc., Cambridge, MA, UK) as an NC. The magnetic bead-antibody complex was washed, re-suspended with 900 μL RIP Wash Buffer, and incubated with 100 μL cell extract overnight at 4 °C. The samples were washed 3 times and placed on magnetic pedestal to collect the magnetic bead-protein complexes. The collected samples and input were separately digested by proteinase K to extract RNA for detecting HOTAIR in subsequent RT-qPCR.

### RNA pull‐down assay

AML cells were respectively transduced with wild-type (WT) biotinylated HOTAIR and mutant (MUT) biotinylated HOTAIR (50 nM each). After 48 h of treatment, the cells were washed with PBS and lysed with specific cell lysis (Ambion, Austin, Texas, USA) for 10 min. After that, the lysate was cultured with M-280 streptavidin magnetic beads (Sigma, St. Louis, MO, USA) pre-coated with RNase-free bovine serum albumin (BSA) and yeast tRNA (Sigma, St. Louis, MO, USA) at 4 °C for 3 h, washed twice with cold lysis buffer, and then washed with the low-salt buffer three times and with high-salt buffer once. Total protein was extracted with high-efficiency RIPA lysate, followed by determination of the level of Dnmt3b using western blot analysis. The experiment was repeated three times independently.

### Chromatin immunoprecipitation (CHIP)

After fixed with 1% formaldehyde, AML cells were treated with ultrasonication. Cells were added with rabbit anti Dnmt3b (ab2851, 1:50, Abcam Inc., Cambridge, MA, UK) to bind to the Dnmt3b-HOXA5 promoter, and then added with Protein A Agarose/Salmon Sperm DNA. The precipitated complex of Dnmt3b antibody-Dnmt3b-HOXA5 promoter was washed and eluted to obtain enriched Dnmt3b-HOXA5 promoter complex. After de-crosslinked, the enriched HOXA5 promoter fragment was purified and subjected to PCR. The experiment was repeated three times independently.

### Flow cytometry

AML Cells were collected, rinsed twice with cold PBS, and suspended with 400 μL 1× Binding Buffer. Afterwards, the cell suspension was incubated with the addition of 5 μL Annexin V-fluorescein isothiocyanate (FITC) at 4 °C for 15 min avoiding exposure to light. Added with 10 μL propidium iodide (PI), the cells were then incubated at 4 °C avoiding exposure to light for 5 min. Finally, the cells were analyzed by flow cytometry (BD FACS Calibur, Becton‑Dickinson, San Jose, CA, USA) within 1 h. The experiment was conducted three times independently.

### 5-ethynyl-2′-deoxyuridine (EdU) assay

The AML cells in the logarithmic growth stage were inoculated into the 24-well plates, with three duplicated wells set in each group. After that, EDU (C10341-1, Guangzhou RiboBio Co., Ltd., Guangzhou, China) was incubated with the cells for 2 h at a final concentration of 10 µmol/L. After removal of the medium, cells were fixed in PBS implemented with 4% paraformaldehyde for 15 min at room temperature, washed twice with PBS conjugated with 3% BSA, incubated with PBS containing 0.5% Triton-100 for 20 min at room temperature, and then rinsed twice with PBS containing 3% BSA. And then, cells in each well were incubated with 100 µL Apollo^®^ 567 (Guangzhou RiboBio Co., Ltd., Guangzhou, China) at room temperature avoiding exposure to light for 30 min, washed twice with PBS containing 3% BSA, and stained with 1× Hoechst 33,342 for 30 min, followed by PBS wash 3 times. After cells were mounted, the number of EDU positive cells as well as the total cells in each field was observed and recorded under a fluorescence microscope (FM-600, Puda Optical Instrument Co., Ltd., Shanghai, China). The positive cells presented red. The experiment was performed three times independently.

### Tumor formation in nude mice

A total of 64 BALB/c nude mice (5–7 weeks old, 18–22 g in weight, purchased from Lingchang Biotech Co., Ltd., Shanghai, China) were selected. The nude mice were housed under the specific pathogen-free (SPF) environment in Animal Experimental Center (Experimental Animal Qualification Certificate No. 257), with comfortable temperature and environment, aseptic feed and water and alternating 12 h light/dark cycles. The AML cell line was infected with lentivirus with a titer of 2 × 10^8^ PFU/mL. After stably infected cell lines were obtained, 0.2 mL cell suspension with a density of 5 × 10^7^ cells/mL was inoculated into the subcutaneous space on the left side of the BALB/c nude mice with 1 mL syringe. A total of 55 mice were implanted with the following different cells for tumor formation with 11 mice in each group: the M-overexpression (oe)-NC (pLV-EGFP-N-NC lentivirus-infected cells), M-oe-HOTAIR (pLV-EGFP-N-HOTAIR lentivirus-infected cells), M-sh-NC (pLKO.1-shRN-NC lentivirus-infected cells), M-sh-HOTAIR (pLKO.1-HOTAIR-shRNA lentivirus-infected cells), and M-oe-HOTAIR + HOXA5 groups (pLV-EGFP-N-HOTAIR + pLV-EGFP-N-HOXA5 lentivirus-infected cells). After inoculation, all nude mice were housed in laminar-flow hoods in the SPF level animal room. After 4 weeks, the mice were euthanized by inhalation of carbon dioxide, and the xenografts were collected and weighed. Tumor measurements of each group were repeated three times. Then tumor tissue was fixed with 10% formalin and embedded in paraffin. Expression of Bax, cleaved-caspase3, p27, and cyclin G were detected by western blot analysis, and EdU assay was conducted to detect cell proliferation. Each experiment was repeated three times.

### Statistical analysis

All data were processed by SPSS 21.0 statistical software (IBM Corp. Armonk, NY, USA). Measurement data were expressed as mean ± standard deviation. Comparisons between two groups were conducted by means of *t*-test, and comparisons among multiple groups were assessed by one-way analysis of variance. A *p* < 0.05 value indicated that the data were statistically significant.

## Results

### AML tissues and cells exhibit upregulated HOTAIR

**Fig. 1:**
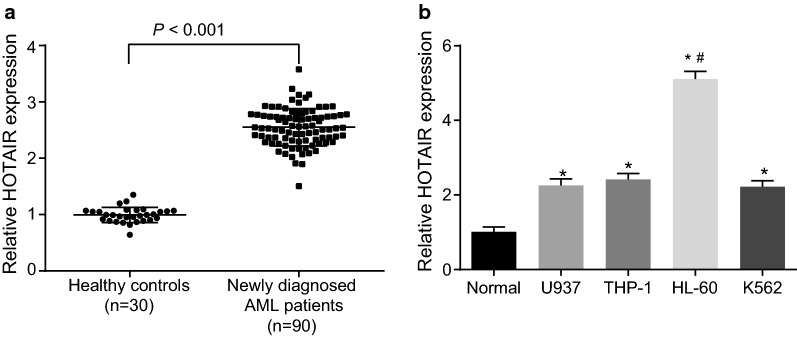
HOTAIR is overexpressed in AML tissues and cells. **a** HOTAIR expression in AML samples examined by RT-qPCR. **b** HOTAIR expression in U937, HL-60, THP-1 and K562 cell lines and normal bone marrow cell line examined by RT-qPCR. *p < 0.05 vs. the normal group; ^#^*p *< 0.05 vs. the U937, THP-1 and K562 cell lines. *RT-qPCR* reverse transcription quantitative polymerase chain reaction, *HOTAIR* Hox transcript antisense intergenic RNA, *NC* negative control, *AML* acute myeloid leukemia. The results were measurement data. Comparisons between two groups were conducted by means of independent sample *t*-test, and comparisons among multiple groups were assessed by one-way analysis of variance. The experiment was independently repeated three times

Initially, the levels of HOTAIR in 90 patients with AML and 30 normal human bone marrow samples were measured. As shown in Fig. 1a, compared with the normal samples, HOTAIR was highly expressed in patients with AML (*p* < 0.001). Then, the HOTAIR level in different leukemia-associated cell lines was determined. The results showed that compared with the hematopoietic stem cells extracted from bone marrow, the expression of HOTAIR was significantly increased in U937, HL-60, THP-1 and K562 cell lines (*p* < 0.05), with HL-60 cell line exhibiting the highest level of HOTAIR (Fig. 1b). Therefore, the HL-60 cell line was selected for subsequent experiments. In conclusion, HOTAIR was upregulated in AML tissues and cells.

### Silencing HOTAIR leads to enhanced apoptosis and repressed proliferation of HL-60 cells

**Fig. 2:**
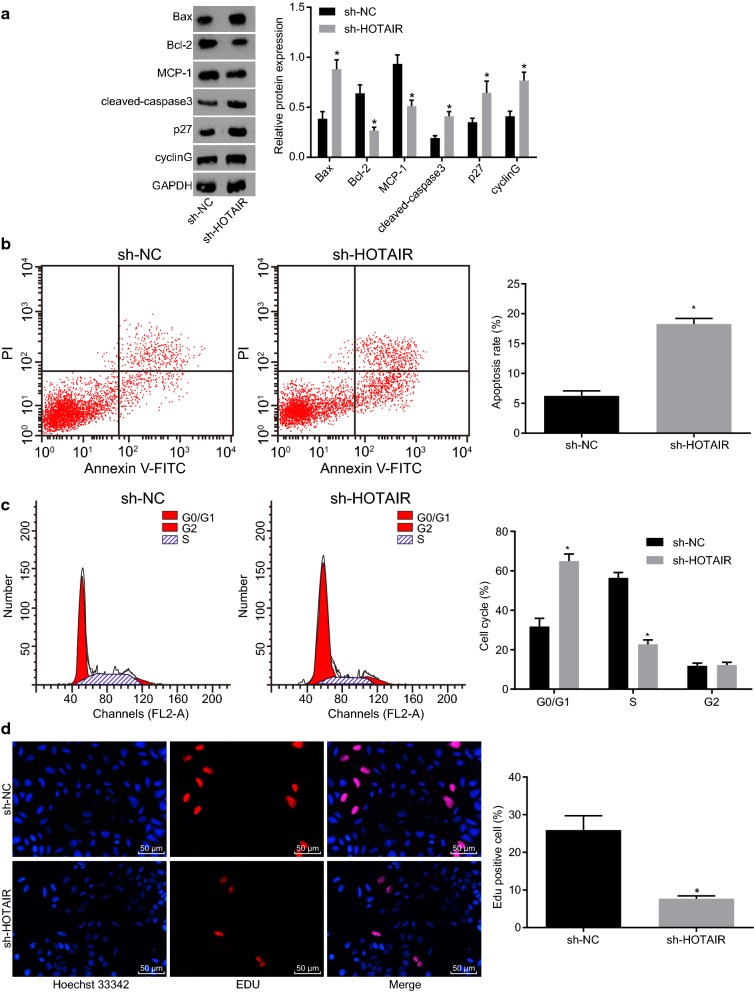
Silencing HOTAIR inhibits proliferation and promotes apoptosis of AML cells. **a** Protein expression of Bax, Bcl-2, MCP-1, cleaved-caspase3, p27 and cyclin G in HL-60 cells after interference with HOTAIR examined by western blot analysis. **b** Cell apoptosis of HL-60 cells after interference with HOTAIR examined by flow cytometry. **c** Cell cycle distribution of HL-60 cells after interference with HOTAIR detected by flow cytometry. **d** The proliferation of HL-60 cells after interference with HOTAIR examined by EdU assay (×200). *p < 0.05 vs. the sh-NC group. *RT-qPCR* reverse transcription quantitative polymerase chain reaction, *HOTAIR* Hox transcript antisense intergenic RNA, *HOXA5* homeobox A5, *EDU* 5-ethynyl-2′-deoxyuridine, *NC* negative control, *AML* acute myeloid leukemia, *Bcl-2* B-cell lymphoma 2, *Bax* Bcl-2 associated X, *MCP-1* monocyte chemoattractant protein 1. The results were measurement data. Comparisons between two groups were conducted by means of independent sample *t*-test. The experiment was independently repeated three times

In order to find out the regulation of HOTAIR on apoptosis and proliferation of HL-60 cells, western blot analysis was conducted for the determination of the protein levels of apoptosis-related factors, including Bax, Bcl-2, MCP-1, cleaved-caspase3, p27, and cyclin G, and flow cytometry and EdU assay were performed to measure cell apoptosis and proliferation, respectively. As shown in Fig. 2a, compared with the sh-NC group, the expression of Bax, cleaved-caspase3, p27 and cyclin G in the sh-HOTAIR group was significantly increased while of MCP-1 and Bcl-2 was decreased (*p *< 0.05). In addition, compared with the sh-NC group, the apoptosis of HL-60 cells was notably increased in the sh-HOTAIR group (*p* < 0.05; Fig. 2b). In comparison with the sh-NC group, the proportion of cells in the G0/G1 phases was elevated while proportion of cells in the S phases was reduced in the sh-HOTAIR group (Fig. 2c). Then, EdU assay revealed that the proliferation of HL-60 cells in the sh-HOTAIR group was notably lower than that in the sh-NC group (*p* < 0.05; Fig. 2d). These results suggested that silencing of HOTAIR promoted apoptosis and inhibited proliferation of AML cells.

### Silencing HOTAIR promotes apoptosis and inhibits proliferation of HL-60 cells by upregulating HOXA5

**Fig. 3:**
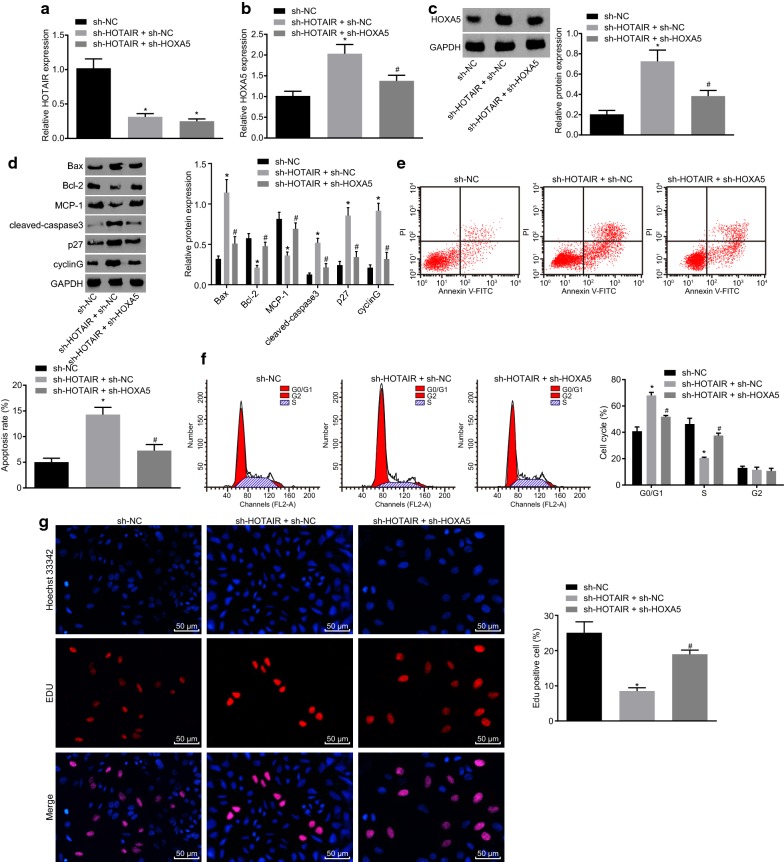
Silencing HOTAIR upregulates HOXA5 to promote apoptosis and suppress proliferation of HL-60 cells. **a** Expression of HOTAIR in HL-60 cells after interference with HOTAIR or HOXA5 detected by RT-qPCR. **b** Expression of HOXA5 at mRNA level in HL-60 cells after interference with HOTAIR or HOXA5 examined by RT-qPCR. **c** Protein expression of HOXA5 in HL-60 cells after interference with HOTAIR or HOXA5 detected by western blot analysis. **d** Expressions of cyclin G, Bax, Bcl-2, MCP-1, cleaved-caspase3, p27 and cyclin G in HL-60 cells after interference with HOTAIR or HOXA5 examined by western blot analysis. **e** Cell apoptosis in HL-60 cells after interference with HOTAIR or HOXA5 examined by flow cytometry. **f** Cell cycle distribution of HL-60 cells after interference with HOTAIR or HOXA5 detected by flow cytometry. **g** Proliferation of HL-60 cells after interference with HOTAIR or HOXA5 examined by EdU assay (×200). *p < 0.05 vs. the sh-NC group; ^#^*p *< 0.05 vs. the sh-HOTAIR + sh-NC group. *RT-qPCR* reverse transcription quantitative polymerase chain reaction, *HOTAIR* Hox transcript antisense intergenic RNA, *HOXA5* homeobox A5, *EDU* 5-ethynyl-2′-deoxyuridine, *AML* acute myeloid leukemia, *Bcl-2* B-cell lymphoma 2, *Bax* Bcl-2 associated X, *MCP-1* monocyte chemoattractant protein 1, *NC* negative control. The results were measurement data. Comparisons among multiple groups were assessed by one-way analysis of variance. The experiment was independently repeated three times

In order to investigate the effect of HOTAIR on HOXA5 expression in HL-60 cells, RT-qPCR was used to detect the expression of HOTAIR after silencing HOTAIR, which showed that the expression of HOTAIR in the sh-HOTAIR + sh-NC group was significantly lower than that in the sh-NC group (*p* < 0.05). In contrast to the sh-HOTAIR + sh-NC group, no obvious difference concerning the expression of HOTAIR was detected in the sh-HOTAIR + sh-HOXA5 group (Fig. 3a). Next, RT-qPCR and western blot analysis were performed to measure the level of HOXA5. As shown in Fig. 3b, c, compared with the sh-NC group, the HOXA5 expression was significantly increased in the sh-HOTAIR + sh-NC group, while decreased in the sh-HOTAIR + sh-HOXA group (*p* < 0.05), suggesting that silencing HOTAIR could upregulate HOXA5. In order to explore the role of HOXA5 in the apoptosis of HL-60 cells, western blot analysis was used to detect the expression of apoptosis-related factors. The results showed that compared with the sh-NC group, the sh-HOTAIR + sh-NC group revealed notably increased expression of Bax, cleaved-caspase3, p27, and cyclin G but decreased expression of MCP-1 and BCcl-2 (*p* < 0.05), while the opposite trend was observed in the sh-HOTAIR + sh-HOXA5 group (*p* < 0.05; Fig. 3d). Then flow cytometry was adopted to assess the effects of HOXA5 on cell cycle distribution and apoptosis of HL-60 cells, which revealed that compared with the sh-NC group, the apoptosis and the proportion of HL-60 cells in the G0/G1 phase were significantly increased, and the proportion of cells in S phase decreased in the sh-HOTAIR + sh-NC group (*p* < 0.05). Compared with the sh-HOTAIR + sh-NC group, the sh-HOTAIR + sh-HOXA5 group showed decreased apoptosis and proportion of cells in G0/G1 phase, but elevated proportion of cells in S phase (*p* < 0.05; Fig. 3e, f). Moreover, EDU was carried out for the detection of the effect of HOXA5 on the proliferation of HL-60 cells. As shown in Fig. 3g, compared with the sh-NC group, the proliferation of HL-60 cells was significantly decreased in the sh-HOTAIR + sh-NC group (*p* < 0.05). When compared with the sh-HOTAIR + sh-NC group, the sh-HOTAIR + sh-HOXA5 group showed notably elevated proliferation of HL-60 cells (*p* < 0.05). The above results demonstrated that silencing HOTAIR promoted the expression of HOXA5, thus promoting apoptosis and inhibiting proliferation of HL-60 cells.

### HOTAIR induces HOXA5 promoter methylation by binding Dnmt3b

**Fig. 4:**
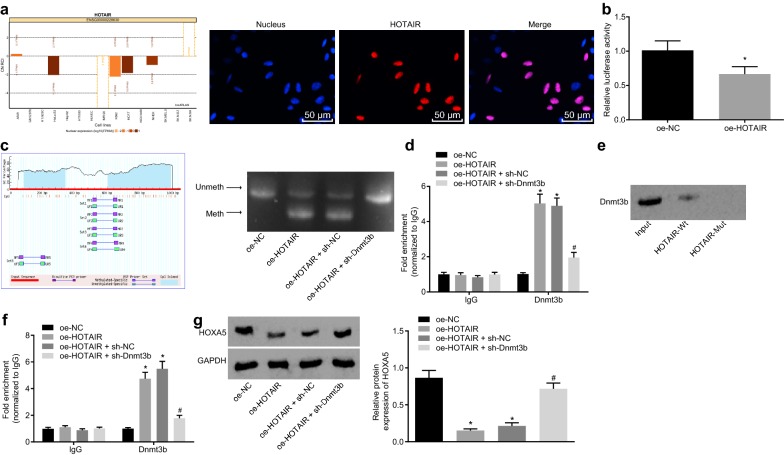
HOTAIR and Dnmt3b downregulates HOXA5 through promoting its methylation. **a** The subcellular localization of HOTAIR predicted by lncATLAS website and verified by FISH (×200). **b** Luciferase assay to confirm the binding of HOTAIR to HOXA5 promoter. **c** Distribution of CpG islands within the HOXA5 promoter region by MethPrimer and methylation level of HOXA5 promoter region detected by MSP. **d** The binding of Dnmt3b to HOTAIR after alteration of HOTAIR or Dnmt3b expression detected by RIP. **e** RNA pull down biotin-labeled HOTAIR and the expression of Dnmt3b in immunoprecipitation examined by western blot analysis. **f** The binding of Dnmt3b with HOXA5 promoter region after alteration of HOTAIR or Dnmt3b expression detected by CHIP-PCR. **g** Expression change of HOXA5 after alteration of HOTAIR or Dnmt3b expression examined by western blot analysis. *p < 0.05 vs. the oe-NC group; ^#^*p *< 0.05 vs. the oe-HOTAIR + sh-NC group. *RT-qPCR* reverse transcription quantitative polymerase chain reaction, *NC* negative control, *FISH* fluorescence in situ hybridization, *RIP* RNA immunoprecipitation, *CHIP* chromatin immunoprecipitation, *HOTAIR* Hox transcript antisense intergenic RNA, *HOXA5* homeobox A5, *Dnmt3b* DNA methyltransferase 3b. The results were measurement data. Comparisons between two groups were conducted by means of independent sample *t*-test, and comparisons among multiple groups were assessed by one-way analysis of variance. The experiment was independently repeated three times

Subcellular localization of HOTAIR was predicated with the LncATLAS website (http://lncatlas.crg.eu/), which uncovered that HOTAIR existed primarily in the nucleus of multiple cell lines. Then FISH was employed to determine the subcellular localization of HOTAIR, finding that HOTAIR was mainly distributed in the nucleus (Fig. 4a). Next, dual luciferase reporter assay was conducted to detect the binding of HOTAIR to HOXA5 promoter. As shown in Fig. 4b, compared with the oe-NC group, the luciferase activity of the oe-HOTAIR group was significantly decreased (*p* < 0.05; Fig. 4b). High methylation level of HOXA5 was once found in AML samples, and HOXA5 methylation has been suggested to play an important role in the occurrence and development of AML [20]. A recent study found that Dnmt3b-mediated DNA methylation acted an essential role in the development of leukemia [21]. So we would like to explore whether HOTAIR and Dnmt3b affected the methylation of HOXA5. CpG islands in HOXA5 promoter region were analyzed via MethPrimer software (https://www.urogene.org) and MS-PCR showed that no methylation occurred at specific sites of HOXA5 in both the oe-NC and oe-HOTAIR + sh-Dnmt3b groups, and methylation existed at specific sites of HOXA5 in both the oe-HOTAIR and oe-HOTAIR + sh-NC groups (Fig. 4c), suggesting that HOTAIR could promote the methylation of HOXA5, which required Dnmt3b. It has also previously revealed that Dnmt3b-mediated DNA methylation was crucial in the progression of leukemia [21]. To further explore how Dnmt3b and HOTAIR function together to regulate HOXA5, RIP was used to detect whether Dnmt3b could bind with HOTAIR. It revealed that compared with the oe-NC group, the combination of HOTAIR and Dnmt3b was significantly increased in the oe-HOTAIR group (*p* < 0.05), and the combination of HOTAIR and Dnmt3b was significantly lower in the oe-HOTAIR + sh-Dnmt3b group than in the oe-HOTAIR + sh-NC group (*p* < 0.05; Fig. 4d). RNA pull down assay was conducted to detect whether HOTAIR affected the degradation of Dnmt3b, and western blot analysis was used to examine the expression of Dnmt3b. It was found that the HOTAIR-Wt group showed decreased degradation of Dnmt3b expression while the HOTAIR-Mut group showed no Dnmt3b (Fig. 4e). Furthermore, CHIP was performed to detect whether Dnmt3b could directly bind to HOXA5 promoter region and whether HOTAIR affected the binding of Dnmt3b with HOXA5 promoter. Compared with the oe-NC group, Dnmt3b was remarkably enriched in HOXA5 promoter region in the oe-HOTAIR group. Compared with the oe-HOTAIR + sh-NC group, the oe-HOTAIR + sh-Dnmt3b group showed reduced enrichment of Dnmt3b in HOXA5 promoter region (*p* < 0.05; Fig. 4f). Western blot analysis showed that compared with the oe-NC group, the expression of HOXA5 in the oe-HOTAIR group was significantly decreased. In contrast to the oe-HOTAIR + sh-NC group, the expression of HOXA5 in the oe-HOTAIR + sh-Dnmt3b group was notably increased (*p* < 0.05; Fig. 4g). Taken together, HOTAIR, together with Dnmt3b, was able to promote the methylation of HOXA5 and then inhibited the expression of HOXA5.

### Silencing HOTAIR promotes apoptosis and represses proliferation of AML cells by elevating HOXA5 in vivo

**Fig. 5:**
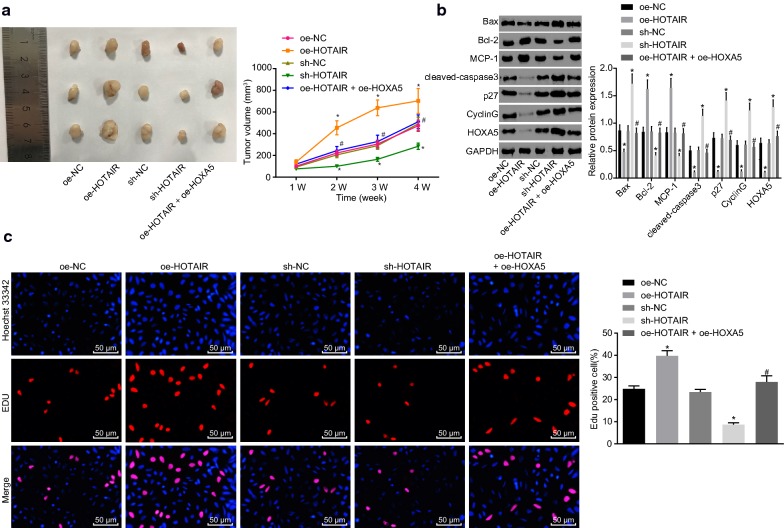
Silenced HOTAIR enhances apoptosis and inhibits proliferation of HL-60 cells in vivo by upregulating HOXA5. **a** The volume of tumor from nude mice transplanted with HL-60 cells overexpressing HOTAIR or HOXA5. **b** Expression of Bax, Bcl-2, MCP-1, cleaved-caspase3, p27 and cyclin G in HL-60 cell overexpressing HOTAIR or HOXA5 detected by western blot analysis. **c** EDU assay to evaluate the proliferation of HL-60 cells after overexpressing HOTAIR or HOXA5 (×200). *p < 0.05 vs. the M-oe-NC and M-sh-NC groups; ^#^*p *< 0.05 vs. the M-oe-HOTAIR and M-sh-HOTAIR groups. *HOTAIR* Hox transcript antisense intergenic RNA, *HOXA5* homeobox A5, *EDU* 5-ethynyl-2′-deoxyuridine, *Bcl-2* B-cell lymphoma 2, *Bax* Bcl-2 associated X, *MCP-1* monocyte chemoattractant protein 1, *NC*negative control. The results were measurement data. Comparisons among multiple groups were assessed by one-way analysis of variance. The experiment was independently repeated three times

In order to study the effects of HOTAIR and HOXA5 on HL-60 cells in vivo, we established xenograft tumor model in nude mice. Compared with the M-oe-NC group, the volume of tumor was significantly elevated in the oe-HOTAIR group while was significantly decreased in the M-sh-HOTAIR group (*p* < 0.05). The volume of tumor in the M-oe-HOTAIR + oe-HOXA5 group was significantly lower than that in the M-oe-HOTAIR group (*p* < 0.05; Fig. 5a). Then the levels of factors related to cell apoptosis were determined using western blot analysis. Figure 5b showed that compared with the M-oe-NC group, the expression of Bax, cleaved-caspase3, p27, cyclin G and HOXA5 was notably reduced while of Bcl-2 and MCP-1 was obviously increased in the M-oe-HOTAIR group (*p* < 0.05). In contrast to the M-sh-NC group, the M-sh-HOTAIR group exhibited increased expression of Bax, cleaved-caspase3, p27, cyclin G and HOXA5 while decreased expression of Bcl-2 and MCP-1 (*p* < 0.05). The expression of Bax, cleaved-caspase3, p27, cyclin G and HOXA5 was notably higher while of Bcl-2 and MCP-1 was significantly lower in the M-oe-HOTAIR + oe-HOXA5 group than those in the M-oe-HOTAIR group (*p* < 0.05; Fig. 5b). Lastly, EDU was conducted to detect the proliferation of HL-60 cells, which revealed that compared with the M-oe-NC group, the cell proliferation in the M-oe-HOTAIR group was significantly elevated (*p* < 0.05). The cell proliferation was significantly reduced in the sh-HOTAIR group compared with the M-sh-NC group (*p* < 0.05). The cell proliferation in the M-oe-HOTAIR + oe-HOXA5 group was significantly lower than that in the M-oe-HOTAIR group (*p* < 0.05; Fig. 5c). The above results suggest that silencing HOTAIR could induce apoptosis and suppress proliferation of AML cells through up-regulating the expression of HOXA5.

### Determination of interference efficiency

The interference efficiency of 3 independent shRAN against HOTAIR was examined. Compared with sh-HOTAIR-1 and sh-HOTAIR-2, the sh-HOTAIR-3 showed the highest silencing efficiency (*p* < 0.05; Additional file 2: Fig. S1). Therefore, subsequent experiments were performed using the sh-HOTAIR-3 to silence HOTAIR.

## Discussion

Despite the continuous improvement of AML treatment, prognosis of patient suffering from AML remains poor, partly due to the high relapse rate and the resistance to chemotherapy [22, 23]. DNA methylation pattern and gene expression profile of AML provides a new understanding in relation to the occurrence and development of AML, ultimately highlighting the potential of new research on novel therapeutic targets [24]. In recent years, HOTAIR, a type of lncRNA, has been demonstrated to participate in the progression of leukemia through regulating DNA methylation, as well as histones [25]. This study explored the potential roles of HOTAIR, HOXA5 methylation and Dnmt3b in AML cells, and got the conclusion that silencing HOTAIR could inhibit the methylation of HOXA5 via recruitment of Dnmt3b and effectively inhibit proliferation and induce apoptosis of AML cells.

Initially, the present study demonstrated that HOTAIR was highly expressed in AML cells. HOTAIR acts as a carcinogenic lncRNA, and elevated HOTAIR was found predict the poor overall survival in patients suffering from leukemia and lymphoma [26]. Moreover, another study has also indicated that HOTAIR is overexpressed in AML tissues [27]. Furthermore, in our study, down-regulating HOTAIR was found to inhibit proliferation and promote apoptosis of AML cells, as supported by elevated Bax, cleaved-caspase3, p27 and cyclin G expressions in the AML cells transfected with sh-HOTAIR. The increased expression of Bax, cyclin D1, and cleaved-caspase3 was related to induced cycle arrest and apoptosis of HL-60 acute leukemia cells [28]. Additionally, a prior study displayed that elevated expression of p27 could decline cell viability and induce cell apoptosis in CD44-treated non-proliferating AML via forced expression of p27Kip1 [29]. Knockdown of HOTAIR triggered by small interfering RNA was previously suggested to suppress the proliferation of HL-60 and K562 cells in AML [12]. Besides, Wu et al. asserted that reduction of HOTAIR could induce apoptosis of K562-R cells in chronic myelogenous leukemia [30]. Furthermore, silencing HOTAIR by small hairpin RNA was found to suppress cell proliferation, induce apoptosis, and decline the colony formation ability in AML patients [31]. Collectively, our results concluded that HOTAIR might be an important target for the treatment of AML.

Additionally, our results found that HOTAIR was attributed to the methylation of the HOXA5 promoter, indicating that the function of HOTAIR in AML might be associated with HOXA5 promoter methylation. As a potential lncRNA biomarker in leukemia, HOTAIRM1 was identified to activate the temporal collinear HOXA gene, including HOXA5 [32]. HOXA5, a HOXA cluster gene, was reported to control the specification of myeloid and erythrocyte lineages, and its constitutive expression was identified to suppress erythropoiesis and induce the production of bone marrow cells [33]. HOTAIR was demonstrated to downregulate HOXA5 in lung cancer [13]. Overexpression of HOTAIR and methylation of HOXA5 were also observed in the progression of breast cancer [19]. A previous study demonstrated that the hyper-methylation of the HOXA5 promoter was related to the arrest of normal cell differentiation, thereby affecting the progression of AML [20]. In specific terms, HOTAIR was observed to promote the methylation of HOXA5 promoter by means of recruiting Dnmt3b. A previous study suggested that HOXA4 has an impact on the overall survival of AML by working with the DNA methylation protein (Dnmt1 or Dnmt3b) [34]. In addition, HOTAIR has been revealed to be correlated to upregulated Dnmt3a and Dnmt3b via the regulation of the DNA methylation in acute leukemia patients, contributing to the induced occurrence of acute leukemia [25]. Moreover, increased binding of Dnmt3b with pre-microRNA-375 (pre-miR-375) promoter could promote the DNA hyper-methylation of pre-miR-375 and decline expression of miR-375, thereby leading to leukemogenesis in AML [35]. Interestingly, our study presented that after overexpression of HOXA5, cell proliferation was decreased and cell apoptosis was increased. Consistent with our results, Xudong Peng et al. observed that HOXA5 inhibited GC cell proliferation in vivo [36]. In addition, another study also found that HOXA5 abrogated cell apoptosis induced by physcion, which is in line with our study [37].

## Conclusion

**Fig. 6:**
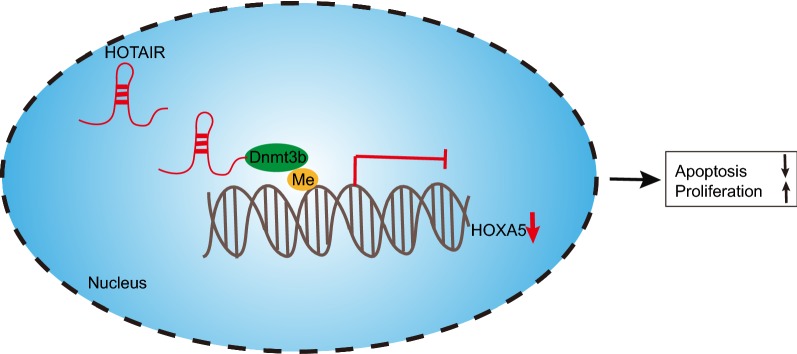
The molecular mechanism that HOTAIR affected the progression of AMI by regulating HOXA5. LncRNA HOTAIR promotes HOXA5 promoter methylation by recruiting Dnmt3b to inhibit expression of HOXA5, thereby promoting the proliferation and inhibiting apoptosis of AML cells. However, silenced could reverse the above development, thus protecting against the deterioration of AML. *HOTAIR* Hox transcript antisense intergenic RNA, *AML* acute myeloid leukemia, *HOXA5* homeobox A5, *Dnmt3b* DNA methyltransferase 3b

In summary, the key findings of the study revealed that HOTAIR was overexpressed in AML and that silencing of HOTAIR repressed methylation of HOXA5 promoter by decreasing Dnmt3b, thereby inhibiting proliferation and inducing apoptosis of AML cells (Fig. 6). Based on the evidence of this study, therapeutic strategies should be directed towards the down-regulation of HOTAIR, which may potentially be a clinically viable target in the treatment of AML.

## Additional files

**Additional file 1: Table S1.** The clinical characteristics of patients.

**Additional file 2: Figure S1.** The interference efficiency of different shRNA against HOTAIR detected by RT-qPCR. *p < 0.05 vs. the sh-NC group; ^#^*p *< 0.05 vs. the sh-HOTAIR-1 and sh-HOTAIR-2 sequences; RT-qPCR, reverse transcription quantitative polymerase chain reaction; HOTAIR, Hox transcript antisense intergenic RNA; NC, negative control. The results were measurement data. Comparisons between two groups were conducted by means of independent sample *t*-test. The experiment was independently repeated three times.

## Abbreviations

AML: acute myelogenous leukemia
FLT3: fetal liver tyrosine kinase 3
ITD: internal tandem duplication
lncRNAs: long non-coding RNAs
HOTAIR: homeobox antisense intergenic RNA
HOXA5: homeobox A5
Dnmt3b: DNA methyltransferase 3b
WHO: World Health Organization
POX: peroxidase
ANAE: alpha-naphthyl-acetate-esterase
CE: specific esterase
PAS: periodic acid-Schiff
RT-qPCR: reverse transcription quantitative polymerase chain reaction
NC: negative control
GAPDH: glyceraldehyde-3-phosphate dehydrogenase
RIPA: radioimmunoprecipitation assay
BCA: bicinchoninic acid
PVDF: polyvinylidene fluoride
BSA: bovine serum albumin
ECL: enhanced chemiluminescence
FISH: fluorescence in situ hybridization
PBS: phosphate-buffered saline
MS: methylation specific
CT: cytosine to thymine
pRL: promoter-renilla luciferase
TK: thymidine kinase
RIP: RNA immunoprecipitation
WT: wild-type
BSA: bovine serum albumin
CHIP: chromatin immunoprecipitation
FITC: fluorescein isothiocyanate
PI: propidium iodide
EDU: 5-ethynyl-2′-deoxyuridine
SPF: specific pathogen-free
