# Supplementary material for: Silencing long non-coding RNA HOTAIR exerts anti-oncogenic effect on human acute myeloid leukemia via demethylation of HOXA5 by inhibiting Dnmt3b
Source: Cancer Cell Int. 2019 Apr 29;19:114. doi: 10.1186/s12935-019-0808-z (PMC6489230; doi:10.1186/s12935-019-0808-z)
Supplement: Supplementary file 1 — Additional file 1: Table S1. The clinical characteristics of patients. [file 12935_2019_808_MOESM1_ESM.docx]

**Additional Table S1** The clinical characteristics of patients

| Clinical characteristics | Cases |
| --- | --- |
| Gender |  |
| Male | 47 |
| Female | 43 |
| Age |  |
| < 45 | 42 |
| ≥ 45 | 48 |
| WBC (× 10^9^/l) |  |
| < 50 | 52 |
| 50 – 99 | 28 |
| > 100 | 10 |
| AML history, n (%) |  |
| De novo | 87 |
| Secondary | 3 |
| High-risk MDS | 0 |
| Cytogenetic risk |  |
| Favorable | 14 |
| Intermediate | 57 |
| Adverse | 12 |
| Undetermined | 7 |
| WHO Performance Status |  |
| 0 | 67 |
| 1 | 14 |
| 2 | 6 |
| 3 | 3 |

Note: WBC, white blood cell; AML, acute myeloid leukemia; MDS, myelodysplastic syndrome; WHO, World Health Organization.
